# Successful Management of Natalizumab-Associated Primary Central Nervous System Lymphoma through Autologous Stem Cell Transplant

**DOI:** 10.3390/curroncol28010022

**Published:** 2020-12-30

**Authors:** Karine Moineau-Vallée, Justine Rinfret, My Hanh Luu Hoai, Valérie St-Louis, France Berthelet, Laurent Létourneau-Guillon, Émilie Lemieux-Blanchard, Alexandre Prat, Jean-Philippe Adam

**Affiliations:** 1 Faculty of Pharmacy, Université de Montréal, Montréal, QC H3T 1J4, Canada; karine.moineau-vallee@umontreal.ca (K.M.-V.); justine.rinfret@umontreal.ca (J.R.); my.hanh.luu.hoai@umontreal.ca (M.H.L.H.); valerie.st-louis@umontreal.ca (V.S.-L.); 2 Department of Pathology, Centre Hospitalier de l’Université de Montréal, Montreal, QC H2X 3E4, Canada; france.berthelet.med@ssss.gouv.qc.ca; 3 Department of Radiology, Centre Hospitalier de l’Université de Montréal, Montreal, QC H2X 3E4, Canada; laurentletg@gmail.com; 4 CHUM Research Center, CHUM, Montréal, QC H2X 3E4, Canada; emilie.lemieux-blanchard.med@ssss.gouv.qc.ca (É.L.-B.); a.prat@umontreal.ca (A.P.); 5 Division of Hematology-Oncology, CHUM, Montréal, QC H2X 3E4, Canada; 6 Department of Neuroscience, Centre Hospitalier de l’Université de Montréal, Montreal, QC H2X 3E4, Canada; 7 Department of Pharmacy, Centre Hospitalier de l’Université de Montréal, Montréal, QC H2X 3E4, Canada

**Keywords:** multiple sclerosis, primary central nervous system lymphoma, natalizumab, autologous hematopoietic stem cell transplant

## Abstract

Natalizumab is used as a second-line treatment for multiple sclerosis (MS). Some reports have linked natalizumab to primary central nervous system lymphoma (PCNSL), although few have described its management. A 45-year-old woman with Balo’s Concentric Sclerosis presented dizziness, vertigo accompanied by dysarthria, weakness on the left side and blurred vision to the right eye after the fourth dose of natalizumab. Magnetic resonance imaging (MRI) and a brain biopsy confirmed the diagnosis of PCNSL. The patient received modified PCNSL chemotherapy (MATRix protocol) followed by high-dose chemotherapy (HDC) supported by an autologous hematopoietic stem cell transplant (ASCT) as a consolidation therapy. Thirty months later, she is still in complete remission of her PCNSL and MS. In this case, whole brain radiotherapy was excluded because it may be associated with an increased risk of neurotoxicity in MS. ASCT was preferred because it has been shown to prevent disability progression in less advanced MS stages. Our patient is the second to receive an ASCT in this context and this option of treatment should be the preferred if the patient is eligible.

## 1. Introduction 

One of the main risk factors for developing primary central nervous system lymphoma (PCNSL) is immunodeficiency [1]. Multiple sclerosis (MS) is an autoimmune disease of the central nervous system that often requires disease modifying therapies (DMT) causing immunosuppression. Epstein–Barr virus (EBV) infection, mostly found in immunosuppressed patients, increases the risk of developing MS and might also be a risk factor of PCNSL [2]. Thus, MS patients are theoretically more at risk of developing PCNSL.

Natalizumab is currently approved in many countries as a second-line treatment for MS. This α4-integrin binding antibody decreases lymphocyte extravasation into the central nervous system (CNS) and small intestine [3]. By decreasing CNS immune surveillance, natalizumab has a potential role in the rapid progression of a pre-existing or new CNS lymphoma [4]. The association between PCNSL and natalizumab is debated. Of the 16 case reports published to date, only a small amount of information on management and outcomes is available [4,5].

In this case report, we discuss PCNSL diagnosis and association with natalizumab, as well as the successful management of the disease through chemotherapy and autologous stem cell transplant (ASCT).

## 2. Case Description

**Figure 1:**
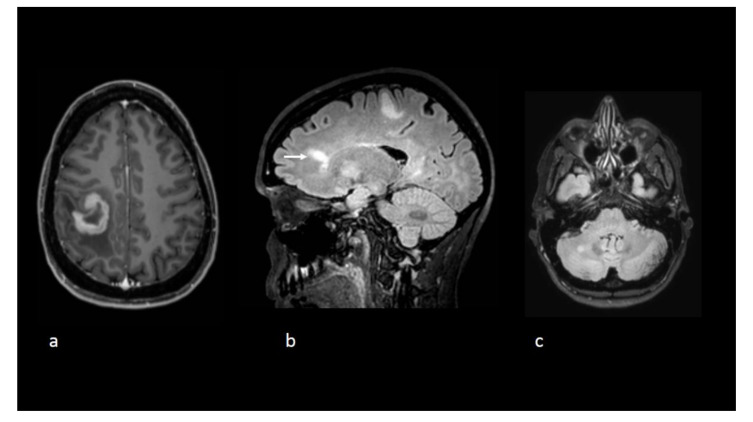
Axial T1-weighted postgadolinium image (**a**) reveals a tumefactive lesion centred on the central sulcus with a thick open-ring enhancement pattern before initiation of natalizumab. Sagittal (**b**) and axial (**c**). Fluid-attenuated inversion recovery (FLAIR) sequences show other nonenhancing lesions including one with a Dawson’s fingers appearance (white arrow).

**Figure 2:**
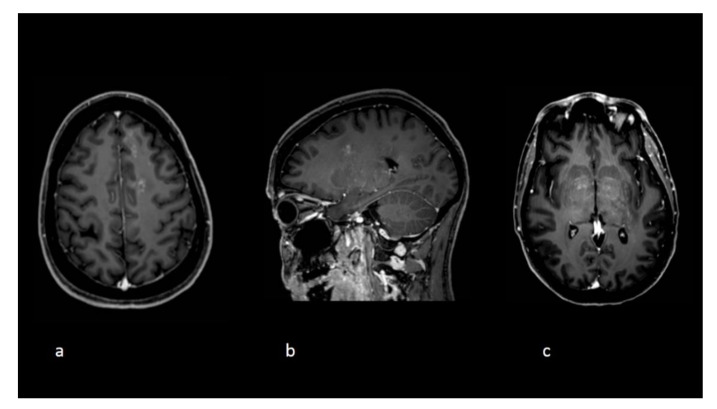
Follow-up imaging at the time clinical deterioration. Axial (**a**,**c**) and sagittal (**b**) T1 postgadolinium images demonstrate new white matter lesions with a punctuate pattern of enhancements and an overall radiological pattern different compared to the initial exams.

**Figure 3:**
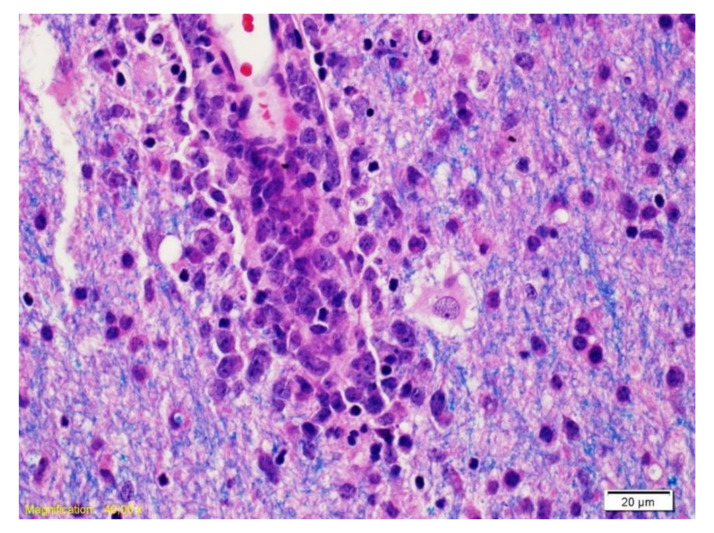
The Luxol Fast Blue coloration combined with hematoxylin/eosin (LFB/H&E) highlights a vessel surrounded by cells with large nucleolus nuclei corresponding to neoplastic B lymphocytes and myelin (blue).

**Figure 4:**
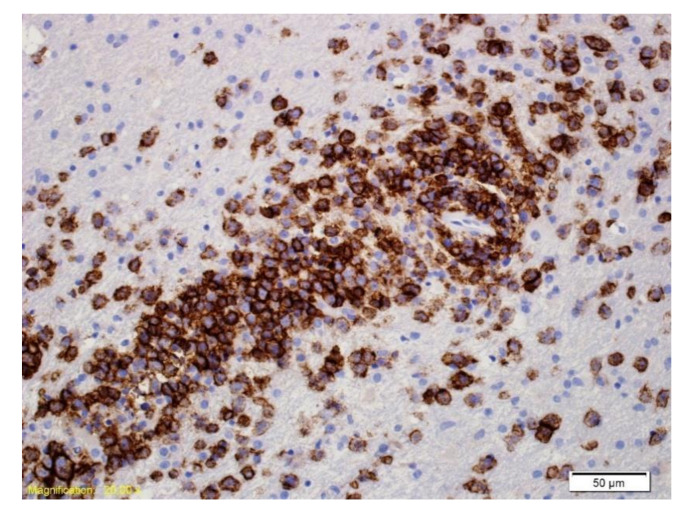
Immunohistochemical labelling with an antibody directed against CD20. The majority of the cells labelled are large neoplastic lymphoid cells.

A 45-year-old Caucasian woman was diagnosed with a variant of MS in 2015, specifically, Balo’s Concentric Sclerosis (BCS). Neuroimaging findings at this time are summarized in Figure 1. Her past medical history included an important episode of optic neuritis in 2015. Following the diagnosis of BCS and negative John Cunningham virus (JCV) screening, the patient was started on natalizumab 300 mg intravenous (IV) every four weeks. Four weeks after the fourth dose of natalizumab, the patient presented dizziness, vertigo accompanied by dysarthria, weakness on the left side and blurred vision to the right eye. Three days later, the patient was admitted for the exacerbation of neurological symptoms. Magnetic resonance imaging (MRI) revealed multiple diffuse subcortical lesions, left more than right, frontal, temporal and in the basal ganglia. At this moment, the suspected diagnosis was more in favor of exacerbation of MS than progressive multifocal leukoencephalopathy (PML) or lymphoma. Microbiology testing was performed and sent out of our health care centre to be analyzed. In the meantime, the patient received methylprednisolone 1 g IV daily for five days and plasma exchange for suspected exacerbation of MS and PML, respectively. On day 3 of hospitalization, the patient showed further neurological deterioration with severe bradycardia and was admitted to the intensive care unit (ICU). The patient’s neurological manifestation was psychomotor retardation, progressive balance disorder with unstable gait, neck stiffness at the end of flexion and paresis of left limbs. A second MRI showed important radiological deterioration (Figure 2). After multiple examinations that excluded other possible diagnoses, a brain biopsy performed on day 6 revealed diffuse CNS B cell lymphoma (Figure 3 and Figure 4). The same day, microbiology results came in with JCV in the cerebrospinal fluid still negative and EBV in the blood positive. On day 8, a “debulking” chemotherapy (rituximab 500 mg/m^2^ and methotrexate 3500 mg/m^2^) was initiated with a significant improvement in her symptoms over the following days. On day 26, she began the MATRix regimen for four cycles every three weeks (see Table 1) and was discharged on day 33 [6]. For consolidation, radiotherapy was not an option because of possible neurotoxicity due to her MS. She received high-dose chemotherapy followed by an autologous stem cell transplant (ASCT) (see Table 1) with a good tolerance. Thirty-five months later, the patient is still in remission of her lymphoma. Furthermore, since the ASCT she has also experienced complete remission of her MS, for which she is no longer receiving treatment. 

## 3. Discussion

Immunosuppression, with or without EBV, is an important risk factor of PCNSL, and the role of natalizumab in the development of PCNSL is debated [3]. Limited data exist to prove this causal relation, because the drug is relatively new, the number of patients reported having experienced PCNSL after drug use are few, and sufficient longitudinal data to review its possible complications with regard to PCNSL development are currently lacking. As an example, three World Health Organization (WHO) cases were reported as unrelated to natalizumab [4]. We report a 45-year-old female (EBV positive and human immunodeficiency virus negative) with a diagnosis of BCS who developed a PCNSL after the fourth cycle of natalizumab. The timing is consistent with the literature where PCNSL was diagnosed after a median of three doses of natalizumab (ranges 1–21) [4]. The patient’s age also matches with other published reports, in which the median age is 44 years old (ranges 28–59) [4]. In our case, there is a probable association between natalizumab and PCNSL based on the Naranjo scale (score of 7) a tool that can help evaluate the probability of the adverse drug reaction [7].

In patients with a PCNSL secondary to natalizumab, the treatment and outcome are unknown for 12 of the 16 reported cases (75%). Among the four remaining cases, two patients who received high-dose methotrexate chemotherapy combined with either intrathecal chemotherapy or radiotherapy died six and 13 months after PCNSL diagnosis, respectively [8,9]. One patient was in remission after surgery and the other after receiving a high-dose methotrexate-based chemotherapy followed by ASCT [5,10]. To our knowledge, this is the second case report describing the successful chemotherapeutic management of natalizumab-associated PCNSL through ASCT.

The MATRix regimen, a combination of cytotoxic agents that crosses the blood-brain barrier, is an option for the initial treatment of PCNSL [6]. Patients with responsive or stable disease after four courses usually proceed with a consolidation therapy consisting of either high-dose chemotherapy followed by ASCT or whole brain radiotherapy (WBRT). In the two largest comparative trial, there were no significant differences in progression-free survival and overall survival at 2 years between WBRT and ASCT; however, the incidence of neurotoxicity is higher with WBRT [11,12]. In this case, WBRT was excluded because it may be associated with an increased risk of neurotoxicity in MS patients [13]. The mechanism of action is not clear, but the hypothesis is that WBRT can cause demyelination to the oligodendrocytes and their precursors, similar to what happens during lesion development in MS [13].

ASCT has been used to treat MS, although there are important risks associated with this procedure, such as mucositis, febrile neutropenia and sepsis [14]. ASCT has been shown to prevent disability progression in most patients, at least for several years, especially in less advanced MS stages [12]. Myeloablative regimens generally used are BEAM or BEAM-like regimens, BuCy-ATG and low intensity regimens, such as fludarabine-based regimens (Flu-Cy or Flu-Mel) [14]. Although our patient did not receive a chemotherapy protocol generally used to treat MS, she received several agents that cross the blood–brain barrier over a longer period to treat PCNSL, including carmustine, which is used in several ASCT protocols to treat MS [14]. Thus, it could explain why our patient experienced remission from her MS.

## 4. Conclusions

Our case contributes to strengthening the possible link between natalizumab and PCNSL, but most importantly details the successful chemotherapeutic management of natalizumab-associated PCNSL through autologous hematopoietic stem cell transplant, as well as complete remission of the MS following ASCT.

## Figures and Tables

**Table 1:** Chemotherapy regimen given to the patient.

Agent	Dose	Posology
MATrix protocol		
Rituximab	375 mg/m^2^	Intravenous (IV) on days −5 and 0
Methotrexate	3500 mg/m^2^	IV on day 1
Cytarabine	2000 mg/m^2^	IV every 12 hours (days 2 and 3)
Thiotepa	30 mg/m^2^	IV on day 4
High-dose chemotherapy before autologous hematopoietic stem cell transplant		
Carmustine	400 mg/m^2^	IV on day −6
Thiotepa	5 mg/kg	IV every 12 hours on days −5 and −4
